# Twitter's Role in Combating the Magnetic Vaccine Conspiracy Theory: Social Network Analysis of Tweets

**DOI:** 10.2196/43497

**Published:** 2023-03-31

**Authors:** Wasim Ahmed, Ronnie Das, Josep Vidal-Alaball, Mariann Hardey, Aïna Fuster-Casanovas

**Affiliations:** 1 Management School Stirling University Stirling United Kingdom; 2 Department of Marketing Audencia Business School Nantes France; 3 Unitat de Suport a la Recerca de la Catalunya Central Fundació Institut Universitari per a la Recerca a l'Atenció Primària de Salut Jordi Gol i Gurina Sant Fruitós de Bages Spain; 4 Health Promotion in Rural Areas Research Group Gerència Territorial de la Catalunya Central Institut Català de la Salut Sant Fruitós de Bages Spain; 5 Faculty of Medicine University of Vic-Central University of Catalonia Vic Spain; 6 Business School Durham University Durham United Kingdom

**Keywords:** COVID-19, coronavirus, Twitter, social network analysis, misinformation, online social capital

## Abstract

**Background:**

The popularity of the magnetic vaccine conspiracy theory and other conspiracy theories of a similar nature creates challenges to promoting vaccines and disseminating accurate health information.

**Objective:**

Health conspiracy theories are gaining in popularity. This study's objective was to evaluate the Twitter social media network related to the magnetic vaccine conspiracy theory and apply social capital theory to analyze the unique social structures of influential users. As a strategy for web-based public health surveillance, we conducted a social network analysis to identify the most influential users in the discussion of the conspiracy, the key websites, and the narratives.

**Methods:**

A total of 18,706 tweets were retrieved and analyzed by using social network analysis. Data were retrieved from June 1 to June 13, 2021, using the keyword *vaccine magnetic*. Tweets were retrieved via a dedicated Twitter application programming interface. More specifically, the Academic Track application programming interface was used, and the data were analyzed by using NodeXL Pro (Social Media Research Foundation) and Gephi.

**Results:**

There were a total of 22,762 connections between Twitter users within the data set. This study found that the most influential user within the network consisted of a news account that was reporting on the magnetic vaccine conspiracy. There were also several other users that became influential, such as an epidemiologist, a health economist, and a retired sports athlete who exerted their social capital within the network.

**Conclusions:**

Our study found that influential users were effective broadcasters against the conspiracy, and their reach extended beyond their own networks of Twitter followers. We emphasize the need for trust in influential users with regard to health information, particularly in the context of the widespread social uncertainty resulting from the COVID-19 pandemic, when public sentiment on social media may be unpredictable. This study highlights the potential of influential users to disrupt information flows of conspiracy theories via their unique social capital.

## Introduction

### Background

Since the World Health Organization declared COVID-19 a global pandemic on March 11, 2020 [1], inconsistent public health messaging has enhanced the appeal of antivaccine and vaccination hesitancy movements on social media [2]. There is already a substantial body of research in the health and social sciences documenting how organized misinformation and disinformation can influence the public's perception of scientific knowledge and public policy [3-6]. It is well established how disinformation led to the overhyped promotion of chloroquine and hydroxychloroquine (drugs used to treat lupus and malaria) as treatments for COVID-19, resulting in market shortages [3]. A randomized controlled trial in the United Kingdom and the United States found that, relative to factual information, exposure to online misinformation about COVID-19 vaccines reduced participants’ intention to vaccinate [7]. Conspiracy theories concerning COVID-19 and vaccinations have substantially altered the landscape of health misinformation and disinformation. Pertwee et al [8] identified an epidemic of uncertainty marked by pervasive social fears and trust issues. Our study investigated a health conspiracy that sprang from web-based disinformation about COVID-19 vaccines that made people magnetic [9,10].

When US-based osteopathic physician Sherri Tenpenny was questioned by Ohio lawmakers on COVID-19 vaccinations in June 2021, she gave a key argument against vaccination, stating that COVID-19 vaccines magnetize people such that “keys can now stick to their forehead” [11]. This magnetic vaccine conspiracy aimed to destabilize and amplify global vaccination skepticism and antivaccine attitudes. The core theme of this disinformation effort was presented as “leaked laboratory” information suggesting that COVID-19 vaccines produce electromagnetic fields at the place of insertion [12]. The wave of manipulated content even showed metal objects affixed to the arms of vaccine recipients [10]. As the popularity of the magnetic vaccine conspiracy continued to take hold, we conducted a social network analysis of Twitter activity.

This study aimed to investigate the Twitter social media network associated with the magnetic vaccine conspiracy theory and analyze social structures by using social capital theory. We used social network analysis for web-based public health surveillance to identify the most influential users in the discussion of the conspiracy, the key websites, and the narratives. Our study especially aimed to answer the following research questions (RQs): how did the social capital of influential users advance public discussion of this conspiracy theory (RQ1), how can network properties and information flow inside this conspiratorial network be identified (RQ2), and how were behavioral messages spread via hashtags (RQ3)?

We used the concept of social capital as a sensitizing concept to emphasize the critical fact that the analytic synergy of web-based and offline networks has allowed for an increase in people's engagement with, interest in, and dissemination of content generated by social media influencers. Our study is likely to be of interest to public health researchers and organizations involved in the surveillance of disinformation across social media.

### Theoretical Foundation: Social Capital and Health Behavior Change

Theoretical models of health behavior change argue that risks to individuals and groups can motivate action and belief in conspiracies [13,14]. Health social media influencers who overstate or entirely fabricate threats tend to amplify conspiracy beliefs [15]. Health conspiracy beliefs are strongly aligned with promoting radical political ideologies and rely on influential users to increase the circulation rate of misinformation and disinformation [16-18].

Recent research has found that social capital, web-based health information, and participation in health communities are strongly linked and are designed to enhance particular beliefs [19]. Social capital refers to human networks and interactions, specifically the social resources available to an individual based on interpersonal ties [20]. In his well-known work related to civic engagement and community in the United States, American political scientist Robert Putnam defined social capital as “connections among individuals – social networks and the norms of reciprocity and trustworthiness that arise from them” [21]. In recent years, new forms of web-based involvement that foster a sense of community among users in sharing misinformation have gained popularity [20,22]. This is due to what some refer to as an “epochal transition” to digital social and cultural forms of connections dominated by bonding and bridging capital [23,24], particularly the types of web-based connections that have been a part of daily life since the COVID-19 pandemic [23,25,26].

The social media communication strategies that make antivaccine content so popular have been studied by scholars attempting to understand health conspiracy theories [27-29]. Bavel et al [30] discovered that influential users were successful at fostering community building to generate a sense of credibility and trustworthiness that helped fuel conspiracist attitudes. According to Latikka et al [31], the rate at which individuals in a community perceive that they receive accurate information may also influence their support for conspiracy theories. Borgonovi and Andrieu [24] argue that a community's stock of social capital is a key factor in spreading health information during the COVID-19 pandemic and changing people's behavior. Previous research has established a correlation between conspiracy ideas and an influencer's increased web-based social capital [32,33]. Two social and behavioral features may account for this association. One is the outcome of a heightened awareness of personal danger, while the other results from a need to feel connected to others. For example, in a study of vaccine conspiracy beliefs conducted by Sallam et al [34], higher hesitancy rates were found among those with lower levels of education who relied on web-based networks to connect to others who shared a similar worldview. Romer and Jamieson [35] found that conspiracy beliefs strengthened the validity views of some major media figures and political leaders who may better meet a conspiracy community's beliefs. These key figures strengthened ties between individuals seeking confirmation of their belief in conspiracy theories. Our study's analytic focus is on ties that are, for the most part, intentionally formed and amplified through multiple acts of propagation in the coconstruction of new health narratives on the internet [36,37]. We argue that such ties are crucial to the formation of conspiracy theories and powerful mechanisms for disseminating factual information. The link between social capital and the dissemination of publicly accessible health information is our primary focus [23].

The primary objective of this study was to demonstrate how influential users can effectively challenge health conspiracy theories. We highlight that influencers can aid public health efforts due to their unique social capital and disseminate truthful and factual information to their followers. Our results contribute to the literature in these areas and offer a theoretical contribution from the application of social capital theory.

## Methods

### Key Terms

This study's *Methods* and *Results* sections use the term *nodes*—a term associated with social network analysis. In our case, the term *node* refers to a Twitter user. In different contexts, the node is the entity under study. The term *edges* refers to the ties and relationships between users, such as retweets, mentions, and replies. Betweenness centrality is a measure of the importance of a node in a network. More precisely, it is a measure of how much an individual node can affect the information flow between other nodes. References are provided to where our study drew upon existing algorithms.

### Stage 1: Data Retrieval

Data were retrieved from 2021 across June 1 to June 13, using the keyword *vaccine magnetic*. This search string was selected because it contains the two keywords that were being used the most to discuss the topic. The aforementioned time period was selected because it falls within a period of time when the conspiracy was shared and discussed the most. This was evidenced by Google Web Trends data showing an elevated interest score for this topic across the dates we examined. We extracted data from a 14-day time period in order to conduct an in-depth, focused investigation and to identify nuances within the data. A total of 18,706 tweets were retrieved via a dedicated Twitter application programming interface. More specifically, the Academic Track application programming interface was used to retrieve data, and the data were analyzed by using NodeXL Pro (version 1.0.1.449; Social Media Research Foundation) [38]. NodeXL Pro was used to look up tweets, using tweet IDs, and NodeXL Pro processed the data into the appropriate format. NodeXL Pro has been previously used to identify influential social network structures in the e-cigarette health scare, antimask campaigns, and 5G COVID-19–related misinformation [27,39,40].

### Stage 2: Data Preparation

Based on the results of the analysis, a total of 22,762 edges (connections between Twitter users) were identified. Using NodeXL Pro, Twitter users were grouped based on their interaction patterns, and the data were preprocessed before being imported into Gephi (version 0.9.2). More specifically, community vertex segmentation was applied in NodeXL by drawing upon the Clauset-Newman-Moore algorithm [41]. A feature of NodeXL allows for the detection of the most used hashtags, URLs, and key influential users.

### Stage 3: Data Analysis

The preprocessed data (from NodeXL, as detailed in the *Stage 2: Data Preparation* section) were exported into Gephi. The nodes were colored based on the community vertex segmentation performed in NodeXL, and the node ranking (size) was determined based on the betweenness centrality metric, which can highlight influential users in a network by identifying vertices that serve as a “bridge” for connecting users and have significant power for information spread within the network. Betweenness centrality scores vary between different networks, and as such, there is not a de facto number that indicates a “high” or “low” score. Instead, betweenness centrality scores were used to rank users from the highest to the lowest in each network. The Force Atlas 2 algorithm was used to lay out the vertices [42].

### Ethics Approval

This study received ethical approval from the institutional review board of the Newcastle University (26055/2022).

## Results

### Results of the Social Network Analysis

**Figure 1:**
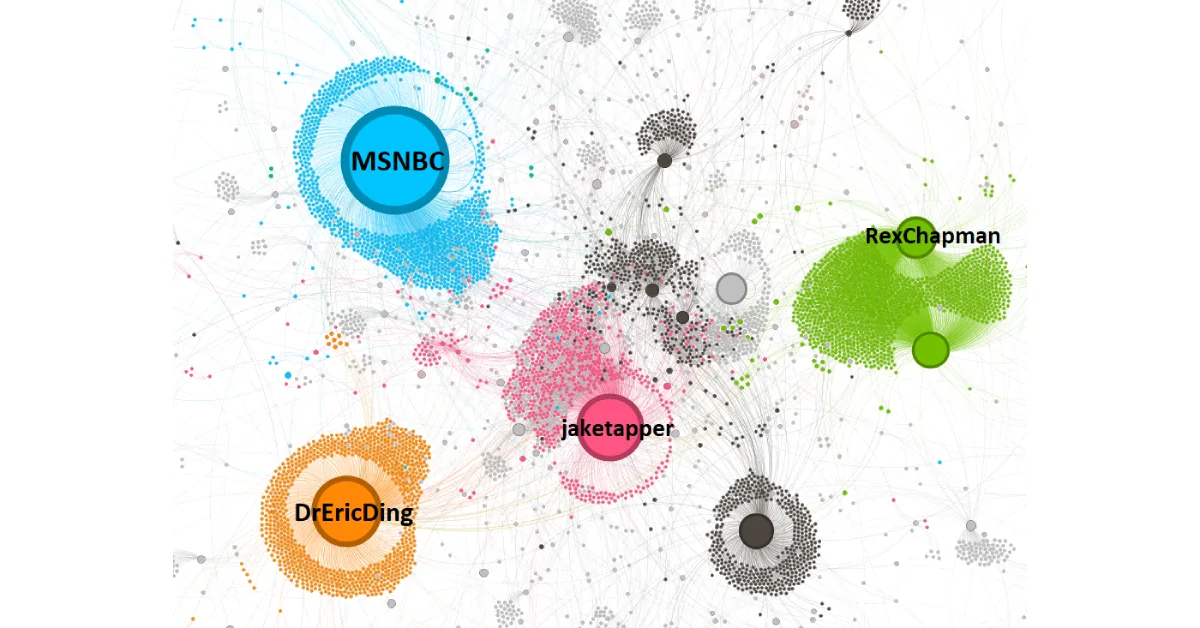
Social network visualization.

Figure 1 shows a graphical representation of the interactions between Twitter users (from June 1 to June 13, 2021) who were conversing about the magnet vaccine conspiracy theory. The circles represent individual Twitter users, and the lines between them represent connections, such as mentions and replies. Different colors represent the different clusters within the network. These groups were found to be discussing different topics related to the conspiracy that vaccines were magnetizing people. The social network analysis results highlight how the discussion revolved around only a handful of key influencers who were driving the discussion and shaping the narrative.

Overall, the hub-and-spoke nature of the conversations indicates that a few key users scattered around the network had the most impact on the network. These key users also had reach outside of their own “cluster.” Table 1 provides an overview of the influential users that are shown in Figure 1. Our study identified 4 key users that were very influential based on their locations within the network as well as their betweenness centrality scores.

Table 1 ranks the most influential users from Figure 1 according to their betweenness centrality and offers further details regarding their information propagation behavior. All users were based in the United States. We have not anonymized the user IDs because these are prominent and well-known social media profile accounts. As Table 1 shows, these accounts advanced public discussion of the conspiracy theory by disproving it, not by supporting it.

Further information on each of the users is provided in the next section.

**Table 1:** Overview of influential users.

Rank	User ID	Betweenness centrality	Followers, n	Impactful tweet
1	MSNBC^a^ (broadcast media account known for political commentary in the United States)	49,565,519	4,407,327	“’This is what anti-vaxxers and conspiracy theorists sound like. It’s all crap. And in pandemic times, it’s actually a danger to public health,’ Brian Williams says on the latest false vaccination conspiracy theory.” [43]
2	DrEricDing (well-known health economist based in the United States)	31,730,774	600,316	“Insanity—Anti-vaccine advocates attempts to prove false claim that #COVID19 vaccines cause magnetism at Ohio legislative committee… by using a *brass* key. Brass = not magnetic by the way. Hence it didn’t go so well. 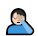 ” [44]
3	jaketapper (media commentator based in the United States)	30,179,783	3,338,717	“Doctor in Ohio makes lunatic claim that vaccines make recipients magnetic” [45]
4	RexChapman (well-known podcaster based in the United States	18,313,395	1,187,905	“People are ‘magnetized’ due to vaccinations. I mean, this is insane.” [46]

^a^MSNBC: Microsoft National Broadcasting Company.

### Results of the Influential User Analysis

Table 1 provides a summary of the influential users that were identified through the social network analysis. Due to the large follower numbers and the disciplines, the top 4 accounts were provided without anonymization. In total, 4 users were specifically selected; based on the social network analysis, they had the highest impact within the network (as shown in Figure 1, these accounts were the most central, with the highest impact).

In Table 1, the *Rank* column is arranged such that users are ordered according to their betweenness centrality scores, which informed each user's raw score. The number of an account's followers is displayed in the fourth column. The *Impactful tweet* column highlights the users’ most influential tweets. Table 1 reveals that MSNBC (Microsoft National Broadcasting Company) was the most followed user on Twitter, with 4,407,327 followers. Table 1, showing the most influential users ranked by importance, highlights how the North American channel MSNBC is in first place in terms of rank, with 4,407,327 followers. An epidemiologist and health economist from Washington, DC, and Virginia are in second place, with 600,316 followers. In third place, with 3,338,717 followers, is an anchor and Washington, DC, correspondent of the American television news network *CNN* (Cable News Network). An ex-basketball player appears in fourth place, with 1,187,905 followers. Most importantly, Table 1 highlights that the most impactful tweets by the top 4 influencers were critical of the conspiracy and were not propagating it.

### Results of the Popular URL Analysis

Table 2 provides an overview of the top 5 most shared websites among users and the frequency with which they were shared.

As shown in Table 2, the most shared website in tweets was a news video from the American television news network *CNN* (683 occurrences), wherein CNN's Jake Tapper and Dr Sanjay Gupta quickly debunked Dr Sherri Tenpenny's affirmation at the Ohio Health Committee that the COVID-19 vaccines caused magnetism [47]. The second most popular website among the tweets (480 occurrences) belongs to the American news website *Raw Story Investigates*. It was reported that Dr Sherri Tenpenny, an antivaccine doctor, informed Ohio legislators that the COVID-19 vaccine magnetizes people [48].

The third most popular website (313 occurrences) also belongs to the *Raw Story Investigates* news website, wherein it was reported that nurse Joanna Oberholt tried to prove Dr Sherri Tenpenny's affirmation and failed [49]. The fourth most popular website belongs to MSNBC’s North American web portal; MSNBC's Brian Williams discussed the conspiracy theory about the magnetization of the COVID-19 vaccines [50]. The fifth most popular website with which people interacted came from India; the corresponding URL [51] leads to a video wherein a man claims to have developed magnetic powers after the second dose of the COVID-19 vaccine.

**Figure 2:**
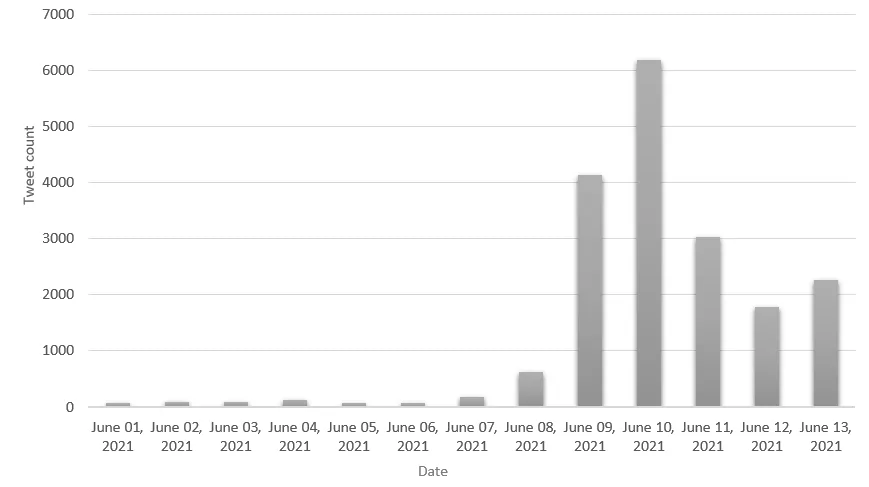
Daily tweet count.

Figure 2 depicts the sequence of tweets, including a single tweet posted on June 8, 2021, by Dr Tenpenny, who indicated at the Ohio State Health Committee that vaccines magnetize individuals. On the same day, the first peak of 620 tweets was observed, and the number of tweets peaked on June 10, with 6180 tweets being sent. Furthermore, it was observed that the conspiracy theory had generated an impact on Twitter, as there was a flow of information during the days after Dr Tenpenny's statement. Figure 2 highlights the impact of the speed of distribution, which strengthens the importance of conspiracy theories in networks and increases the number of influential users seeking to propagate conspiracy claims.

Figure 2 provides an overview of the daily number of tweets.

**Table 2:** Overview of the most shared websites.

Rank	Titles	Occurrences, n
1	Tapper and Gupta react to Doctor's unhinged vaccine claim [47]	683
2	Anti-vaxxer tells Ohio lawmakers that the COVID-19 vaccine magnetises people: 'Put a key on their forehead -- it sticks' [48]	480
3	WATCH: Ohio nurse flops trying to prove anti-vax Doctor's bizarre magnet conspiracy theory [49]	313
4	Conspiracy theorists think Covid vaccine makes you magnetic [50]	302
5	Ulhasnagar man claims developing magnetic powers after taking second dose of Covid-19 vaccine [51]	302

### Results of the Hashtag Analysis

Table 3 highlights the most used hashtags in the tweets.

As shown in Table 3, the most used hashtag was *#covid19*, which was used 1194 times by users in the period studied. Far from the first, the second most used hashtag was *#vaccine*, which was used 354 times. The last in the ranking was *#covidvaccine*, which was used 83 times by users. The hashtags *#demvoice1* and *#wtpblue* ("We the People Blue") are both used by Democratic aligned grassroots communities in the United States, indicating that the topic was circulated within politically engaged, Democratic leaning communities. Interestingly, the hashtag *#magneto* was also used, which refers to a fictional character from American comic books who also appears in the *X-Men*; one of Magneto's powers is to draw upon magnetism. The perceived threat to individuals was clearly drawn from connecting health fears (hashtags 1, 2, 4, and 10 in Table 3) and from potential health activism (hashtags 8 and 9 in Table 3).

**Table 3:** Overview of the most popular hashtags.

Rank	Top hashtags	Count, n
1	*#covid19*	1194
2	*#vaccine*	354
3	*#demvoice1*	151
4	*#magnetic*	135
5	*#wtpblue*	115
6	*#magneto*	104
7	*#science*	91
8	*#vaccinessavelives*	88
9	*#thisisourshot*	86
10	*#covidvaccine*	83

## Discussion

### Principal Findings

For RQ1, we identified the influential users within the network and found that they were actively tweeting against the conspiracy. It is important to emphasize that among these influential users, there were a large number of users who were not associated with health but were associated with politicians and their vast networks. Our study reveals that a network of mainstream media, important health clinicians, and notable public people were the first to educate the public about the nature and dangers of the Twitter-circulating vaccine magnetization conspiracy [39]. Other research [52], such as research examining discussions around blood clot risk related to vaccines, has also found that important health clinicians are critical in disseminating factual information to the public by using social media.

All of the influential users in this study were located in North America. Each influencer has their own unique Twitter followers that respect and rely on their thoughts. These findings contribute to the literature on health communities emphasizing the significance of studying web-based social capital as people's networks and affiliations extend increasingly beyond the physical realm. Moreover, previous research has found that conspiratorial beliefs tend to lend credibility to purposely provocative social media identities whose diverse interests differ from those of the average person [35].

The outcomes of our social network study demonstrate how influential users acted as effective broadcasters against the vaccine magnetization conspiracy, extending the reach of anticonspiracy messages beyond their own Twitter followers (RQ2). The bulk of these significant networks resembled broadcast networks in that they not only helped to broaden the reach of anticonspiracy messages but also amplified them for broader public education.

According to our study, the topic of magnetic vaccines attracted significant engagement worldwide. Based on the pseudoscientific arguments presented by a number of conspiracy theorists [27], few users felt that the conspiracy had scientific underpinnings. The hashtags *#magnetic* and *#magneto* were used 239 times in total (*#magnetic*, n=135; *#magneto*, n=104), ranking fourth and sixth, respectively, among all hashtags in the dataset; *#magnetic* was the most used hashtag referring to the subject of the conspiracy itself. Our findings underscore the necessity of conducting social network analysis as a public health surveillance technique for the discovery of sources of misinformation on social networks. Indeed, with regard to RQ3, the account that had the highest levels of trustworthiness within the network was the North American television channel MSNBC. This was followed by an ex-basketball player. With regard to the most shared websites, it is notable that only one was neutral toward the conspiracy (ranked as number 5 in Table 2). The other popular shared websites disproved the conspiracy, and some of them mocked the idea of people becoming magnetized after COVID-19 vaccination. However, it is interesting to note that any single URL did not appear to be used widely or significantly within tweets, indicating that users were drawing upon a variety of web sources.

Previous research has highlighted the role of influential users in disseminating misinformation [27]. In contrast, our study highlights the roles of influential users and social capital in counterpromoting pseudoscience logic while educating the wider population during a global public health crisis. Our study complements research that has highlighted the positive aspects of social media in, for instance, raising awareness of vaccination safety [53]. Governments, humanitarian agencies, and policy makers can learn 2 important lessons from this study. First, it is often difficult to detect underlying pseudodisinformation movements that occur underneath a bigger anti–health care agenda, such as wider antivaccine propaganda. Niche misinformation search and network analyses should be ongoing in order to capture and diffuse networks of this character. Second, another effective combat strategy is using a network of mainstream media and influential public figures with high social capital who can identify, label, and amplify these misinformation campaigns within their extended network, thereby enhancing public awareness during a health care crisis.

### Conclusion

Although social capital is comprised of both conscious elements and unconscious elements, the study of the emergence and evolution of health conspiracies, with its emphasis on radical uncertainty and discovery, places greater emphasis on the implicit and trust-based dimension of social capital—the bridging of social capital and the manner in which social capital is amplified on the internet. This research highlights the susceptibility of vulnerable communities to conspiracy theory messaging and the possibility of new anxieties regarding the effects of vaccination in the future. Social media users' perceived social capital can be positively leveraged in order to mitigate disinformation campaigns.

## Abbreviations

CNN: Cable News Network
MSNBC: Microsoft National Broadcasting Company
RQ: research question
