# Correction: Twitter’s Role in Combating the Magnetic Vaccine Conspiracy Theory: Social Network Analysis of Tweets

**DOI:** 10.2196/113278

**Published:** 2026-10-20

**Authors:** Wasim Ahmed, Ronnie Das, Josep Vidal-Alaball, Mariann Hardey, Aïna Fuster-Casanovas

**Affiliations:** 1 Stirling Management School University of Stirling Stirling United Kingdom; 2 Department of Marketing Audencia Business School Nantes France; 3 Unitat de Suport a la Recerca de la Catalunya Central Fundació Institut Universitari per a la Recerca a l'Atenció Primària de Salut Jordi Gol i Gurina Sant Fruitós de Bages Spain; 4 Health Promotion in Rural Areas Research Group Gerència Territorial de la Catalunya Central Institut Català de la Salut Sant Fruitós de Bages Spain; 5 Faculty of Medicine University of Vic - Central University of Catalonia Vic Spain; 6 Durham University Business School Durham University Durham United Kingdom

In “Twitter’s Role in Combating the Magnetic Vaccine Conspiracy Theory: Social Network Analysis of Tweets” [1], the authors have identified a number of wording and typographical errors. The manuscript was written by a multinational team working across Spain, France, and the United Kingdom, and several of the errors originated in drafting by colleagues writing in English as a second language and were missed at proof. The authors wish to correct the record. None of the changes affect the data, the analysis, or the conclusions of the study.

In the Objective section of the Abstract, the following sentence was revised:

As a strategy for web-based public health surveillance, we conducted a social network analysis to identify the important opinion leaders sharing the conspiracy, the key websites, and the narratives.

The sentence now reads:

As a strategy for web-based public health surveillance, we conducted a social network analysis to identify the most influential users in the discussion of the conspiracy, the key websites, and the narratives.

In the Background section of the Introduction (first paragraph), the following sentence was revised:

It is well established how disinformation led to the overhyped promotion of chloroquine and hydroxychloroquine (drugs used to treat lupus and malaria) as treatments for COVID-19, resulting in market shortages [5].

The sentence now reads:

It is well established how disinformation led to the overhyped promotion of chloroquine and hydroxychloroquine (drugs used to treat lupus and malaria) as treatments for COVID-19, resulting in market shortages [3].

Reference 5 remains cited in the sentence before.

In the same section (third paragraph), the following sentence was revised:

We used social network analysis for web-based public health surveillance to identify the key opinion leaders sharing the conspiracy, the key websites, and the narratives.

The sentence now reads:

We used social network analysis for web-based public health surveillance to identify the most influential users in the discussion of the conspiracy, the key websites, and the narratives.

In the same paragraph, the following phrase was revised:

how did the social capital of influential users promote the spread of this conspiracy theory

The phrase now reads:

how did the social capital of influential users advance public discussion of this conspiracy theory

In the fourth paragraph of the same section, the following sentence was revised:

Our study is likely to be of interest to public health researchers and organizations involved in the survivance of disinformation across social media.

The sentence now reads:

Our study is likely to be of interest to public health researchers and organizations involved in the surveillance of disinformation across social media.

In the first paragraph of the Background section in the Introduction, the following sentence was revised:

In another investigation of a randomized controlled experiment in the United Kingdom and the United States on the effect of misleading information concerning COVID-19 on people’s vaccination intentions, it was observed that people’s vaccination intentions decreased after receiving at least one type of false information [7].

The sentence now reads:

A randomized controlled trial in the United Kingdom and the United States found that, relative to factual information, exposure to online misinformation about COVID-19 vaccines reduced participants’ intention to vaccinate [7].

The following reference has been added as reference 7, with all subsequent references renumbered accordingly.

7. Loomba S, de Figueiredo A, Piatek SJ, de Graaf K, Larson HJ. Measuring the impact of COVID-19 vaccine misinformation on vaccination intent in the UK and USA. Nat Hum Behav. 2021 Mar;5(3):337-348. doi: 10.1038/s41562-021-01056-1.

In the same paragraph, the following sentence was revised:

Pertwee et al [7] identified a pandemic of uncertainty marked by pervasive social fears and trust issues.

The sentence now reads:

Pertwee et al [8] identified an epidemic of uncertainty marked by pervasive social fears and trust issues.

In the Theoretical Foundation: Social Capital and Health Behavior Change section of the Introduction (second paragraph), the following sentence was revised:

In his well-known study of the impact of social capital on economic security, development, and political participation, American political scientist Robert Putnam identified social capital as a critical factor in these areas, allowing for “connecting among individuals...social networks and norms of reciprocity and trustworthiness that arise from them” [20].

The sentence now reads:

In his well-known work related to civic engagement and community in the United States, American political scientist Robert Putnam defined social capital as “connections among individuals – social networks and the norms of reciprocity and trustworthiness that arise from them” [21].

The quotation had been cited to Shomron et al, which is a different work, and the original source was absent from the reference list. The following reference has been added as reference 21, with all subsequent references renumbered accordingly:

21. Putnam RD. Bowling Alone: The Collapse and Revival of American Community. New York, NY: Simon and Schuster; 2000:19. [ISBN: 978-0-7432-0304-3]

In the Stage 3: Data Analysis section of the Methods, the following sentence was revised:

The Force Atlas 2 algorithm was used to lay out the vertices [39].

The sentence now reads:

The Force Atlas 2 algorithm was used to lay out the vertices [42].

Reference 39 is the community detection algorithm of Clauset, Newman, and Moore, which remains correctly cited earlier in the same section. The source for Force Atlas 2 had been omitted and has been added as reference 42, with all subsequent references renumbered accordingly:

42. Jacomy M, Venturini T, Heymann S, Bastian M. ForceAtlas2, a continuous graph layout algorithm for handy network visualization designed for the Gephi software. PLoS One. 2014 Jun 10;9(6):e98679. [doi: 10.1371/journal.pone.0098679] (PMID: 24914678)

In the Results of the Social Network Analysis section of the Results (third paragraph), the following sentence was revised:

We have not anonymized the user IDs because these are prominent and well-known social media profile accounts that, we argue, are used to promote health conspiracy theories.

The sentence now reads:

We have not anonymized the user IDs because these are prominent and well-known social media profile accounts. As Table 1 shows, these accounts advanced public discussion of the conspiracy theory by disproving it, not by supporting it.

In the Results of the Influential User Analysis section of the Results (second paragraph), the following phrase was revised:

an anchor and Washington, DC, correspondent of the American magazine CNN (Cable News Network)

The phrase now reads:

an anchor and Washington, DC, correspondent of the American television news network CNN (Cable News Network)

In the Results of the Popular URL Analysis section of the Results (second paragraph), the following phrase was revised:

a news article from the American magazine CNN (683 occurrences)

The phrase now reads:

a news video from the American television news network CNN (683 occurrences)

In the same paragraph, the following phrase was revised:

belongs to the American magazine Raw Story Investigates

The phrase now reads:

belongs to the American news website Raw Story Investigates

In the third paragraph of the same section, the following phrase was revised:

also belongs to Raw Story Investigates magazine

The phrase now reads:

also belongs to the Raw Story Investigates news website

In the Results of the Hashtag Analysis section of the Results (second paragraph), the following sentence was revised:

The hashtag #demvoice1 belongs to followers of America’s democratic parties, and the hashtag #wtpblue belongs to conservative political parties in America, indicating that the debate may have become politicized.

The sentence now reads:

The hashtags #demvoice1 and #wtpblue (“We the People Blue”) are both used by Democratic aligned grassroots communities in the United States, indicating that the topic was circulated within politically engaged, Democratic leaning communities.

In the Principal Findings section of the Discussion (fourth paragraph), the following sentence was revised:

The keywords magnetic and magneto were referenced 149 times as hashtags, making them the most associated and relevant topics in the context of #vaccine on this network.

The sentence now reads:

The hashtags #magnetic and #magneto were used 239 times in total (#magnetic, n=135; #magneto, n=104), ranking fourth and sixth, respectively, among all hashtags in the dataset; #magnetic was the most used hashtag referring to the subject of the conspiracy itself.

In the same paragraph, the intended reference is to Table 2, which lists the most shared websites. Table 1 has only four entries. The following phrase was revised:

only one was neutral toward the conspiracy (ranked as number 5 in Table 3)

The phrase now reads:

only one was neutral toward the conspiracy (ranked as number 5 in Table 2)

The Conflicts of Interest section was revised from the following:

None declared.

The section now reads:

Reviewer Francesc Lopez-Segui had previously published with members of the author group. The handling editor determined that the content of the review did not unduly influence the decision to accept the article.

The correction will appear in the online version of the paper on the JMIR Publications website, together with the publication of this correction notice. Because this was made after submission to PubMed, PubMed Central, and other full-text repositories, the corrected article has also been resubmitted to those repositories.
